# Clinical characteristics, predisposing factors, and management of moraxella keratitis in a tertiary eye hospital

**DOI:** 10.1186/s12348-024-00417-x

**Published:** 2024-07-30

**Authors:** Mohammad Soleimani, Sadra Jalali Najafabadi, Alireza Razavi, Seyed Ali Tabatabaei, Saeed Mirmoosavi, Hassan Asadigandomani

**Affiliations:** 1 Ocular Trauma and Emergency Department, Farabi Eye Hospital, Tehran University of Medical Sciences, Qazvin Square, Qazvin Street, Tehran, Iran; 2 Research Center for Clinical Virology, Tehran University of Medical Science, Tehran, Iran; 3 Endocrinology and Metabolism Research Center (EMRC), Vali-Asr Hospital, Tehran University of Medical Sciences, Tehran, Iran

**Keywords:** Keratitis, Corneal perforation, Moraxella keratitis, Prognosis

## Abstract

**Purpose:**

The Moraxella species is a very uncommon pathogen that leads to microbial keratitis (MK). This study aimed to evaluate the clinical features, predisposing factors, and outcomes of Moraxella keratitis in patients of a tertiary eye hospital.

**Methods:**

This retrospective study was conducted from 2015 to 2022, on patients who were admitted with the diagnosis of Moraxella keratitis confirmed by positive culture in a referral eye hospital. Demographics, predisposing factors, best-corrected visual acuity (BCVA), and prognosis were assessed.

**Results:**

A total of 106 individuals diagnosed with Moraxella keratitis, were analyzed. The mean age was 54.42 ± 19.43 years. The mean baseline BCVA of the patients was 2.28 ± 0.6 LogMAR, while this amount reached 1.49 ± 0.81 in the 6-month follow-up (P-value = 0.02). The mean BCVA in the six-month follow-up of the patients who needed surgical interventions was significantly lower than the patients who received only medical treatment (2.15 ± 0.65 vs. 1.29 ± 0.75 LogMAR, P-value = 0.02). Patients with diabetes and those without diabetes did not substantially vary in the prevalence of corneal perforation (P-value = 0.515). Three predisposing factors including corneal perforation (odds ratio = 19.27, P-value = 0.001), hypertension (HTN) (odds ratio = 3.62, P-value = 0.03), and older age (odds ratio = 1.03, P-value = 0.008) were significantly associated with more need for surgical interventions.

**Conclusion:**

In this cohort, poor prognosis necessitating surgical interventions in Moraxella keratitis was found to be associated with corneal perforation, HTN, and older age.

## Background

Corneal opacity is a significant contributor to global blindness, ranking fifth in prevalence and accounting for approximately 3.2% of all cases [1]. According to a recent report by the World Health Organization (WHO), approximately 6 million people around the world suffer from cornea-related blindness or moderate to severe visual impairment [1]. It is estimated that corneal opacity is responsible for 1.5-2.0 million cases of unilateral blindness annually. This highlights a significant and unchecked burden on human health that needs to be addressed with urgency [2, 3]. Corneal opacity with visual impairment can result from infections, trauma, inflammation, degeneration, or nutritional deficiencies [1, 4]. Globally, infectious keratitis is the most common cause of corneal blindness, both developed and developing [5].

Microbial keratitis (MK) is a severe ocular infection that poses a significant threat to vision. Timely diagnosis and intensive topical antimicrobial therapy are essential to preserve the patient’s sight [6–9]. The incidence of Moraxella keratitis (MK) is significantly impacted by location and population, ranging from 6 to 52 per 100,000 in ‘Western’ nations such as the United Kingdom (UK), the United States of America (USA), and Australia in 2012 [10]. The South Asian region has been reporting a high incidence of MK, with as many as 113 cases per 100,000 reported in southern India and 799 cases per 100,000 in Nepal [11]. Many patients require hospitalization to ensure strict adherence to intensive topical antimicrobial therapy and to facilitate close monitoring. However, there is a shortage of cost evaluations of MK in the international literature, with only a limited number of studies conducted in countries such as the UK, the US, Taiwan, and India [11–15]. Between 2013 and 2018, the mean expense of procedural treatment for a patient with MK was confidently estimated with confidence to be $USD 1788.7 in the US and $USD 1788.7 in the UK [12, 14].

Moraxella species are a well-established cause of bacterial keratitis, particularly in individuals who are immunocompromised or have a history of chronic alcoholism [16]. Moraxella infections can cause severe damage to the eyes, ranging from minor blepharoconjunctivitis [17] to stromal involvement with corneal perforation [18]. Moraxella keratitis has been associated with some ocular and systemic risk factors. Contact lens wearing, blepharitis, previous ocular surgery, thyroid eye disease, ocular trauma, and herpes simplex keratitis are local risk factors, whereas immunocompromised patients, alcohol dependence, old age, thyroid disease, and diabetes mellitus are systemic risk factors [19–22]. In different regions and at different times, there may be different predisposing risk factors for Moraxella keratitis. Similarly, clinical findings and treatment outcomes may differ by area. This study aimed to evaluate the clinical features, predisposing factors, and outcomes of Moraxella keratitis in patients of Farabi Eye Hospital from 2015 to 2022.

## Methods

### Study design and setting

This is a retrospective study performed on patients admitted with the diagnosis of Moraxella keratitis proven by positive culture in Farabi Eye Hospital, Tehran, Iran, from 2015 to 2022. Patient records were reviewed for demographic data, past medical history, keratitis symptoms and signs, predisposing factors, antibiotic sensitivity, treatments (medications and surgical interventions), and follow-up data. The size, shape, location of corneal infiltrations, and presence of hypopyon were recorded. The patient’s cornea samples were incubated in a chocolate blood agar culture medium at 35 °C. Bacteria identification was accomplished through the use of gram staining as well as biochemical tests. While cultivating bacteria, direct microscopic observation was conducted simultaneously. To ensure that the Moraxella isolates were the causative pathogens of keratitis and not contaminants, cultures were taken from the affected eye on multiple occasions (at least twice). Consistent isolation of Moraxella from these repeated cultures supported its role as the causative pathogen. Antibiotic susceptibility was assessed using the disc diffusion method.

Indications for admission of the patients included the involvement of the central part of the cornea, infiltration size above 2 mm, the presence of corneal thinning or perforation, and the inability of the patient to refer for regular daily follow-up. For 48 h, the initial antibiotic prescription protocol consisted of 1 drop per hour, which was adjusted according to clinical response and bacterial susceptibility. Patients were visited daily by a well-experienced cornea specialist, and in case of non-response to medication, surgical interventions including cyanoacrylate glue application or therapeutic penetrating keratoplasty (PKP), were performed.

Patients’ visual acuity was calculated according to Snellen’s chart and converted to LogMAR for related analyses. In cases where visual acuity was less than 1/10, count finger, hand motion, light perception (LP), and no light perception (NLP) values were used. According to the study of Day et al. [23], the values of 2.1, 2.4, 2.7, and 3 LogMAR were considered for visual acuities of count finger, hand motion, LP, and NLP, respectively.

### Ethical consideration

This study was performed after permission from the Ethics Committee in Biomedical Research of Tehran University of Medical Sciences (Code: IR.TUMS.FARABIH.REC.1400.074). Written consent was obtained from each patient to add data to the database. Patient identifiers were coded and removed to ensure privacy and confidentiality. The data was only accessible to the researcher and the data collectors.

### Statistical analysis

Continuous variables were assessed for normality using the Kolmogorov-Smirnov and Shapiro-Wilk tests. If normality could be assumed, the data was reported as mean ± standard deviation (SD); otherwise, it was reported as median ± interquartile range (IQR). Qualitative values were reported as frequency (percentage). Due to the non-parametric nature of the data, we used the Mann-Whitney test to compare the means between groups. Fisher’s exact test and the Chi-square test were utilized to compare categorical variables. A multinomial logistic regression was used to predict a nominal outcome variable with more than two categories that lack a given rank or order. For data analysis, the Statistical Package for the Social Sciences (SPSS Inc., version 26.0, Chicago, IL, USA) was used at a significance level of 0.05.

## Results

A total of 106 individuals with the diagnosis of Moraxella keratitis, were included from 2015 to 2022. The mean age was 54.42 ± 19.43 years (range 6–96 years). Other demographic and clinical characteristics of the patients are summarized in Table 1 (Table 1).

**Table 1:** Baseline demographics and clinical characteristics of study participants (**HTN;** hypertension)

Variables	Sub-groups	Number (percentage%)
Gender	*Male*	87 (82.1)
	*Female*	19 (17.9)
Age (year)	*< 50*	50 (47.2)
	*> 50*	56 (52.8)
Comorbidities	*Diabetes*	20 (18.9)
	*Immunodeficiency*	5 (4.7)
	*Opium consumption*	18 (17)
	*HTN*	24 (22.6)
	*Hyperthyroidism*	3 (2.8)
	*Hypothyroidism*	5 (4.7)
	*Exposure Keratopathy*	14 (13.2)
	*Corneal perforation*	18 (17)
Past surgery type	*Tarsorrhaphy*	3 (2.8)
	*Penetrating Keratoplasty*	11 (10.4)
	*Phacoemulsification*	2 (1.9)
	*Other*	13 (12.3)
	*None*	77 (72.6)

At the time of presentation, the sizes of infiltrations were categorized into three groups: less than 3 mm, 3 to 6 mm, and greater than 6 mm. The infiltrate measured less than 3 mm in 15 eyes (14.2%), between 3 and 6 mm in 69 eyes (65.1%), and more than 6 mm in 22 eyes (20.7%). Hypopyon was observed in 53 eyes (50%) during the initial examination. Four patients (3.8%) experienced endophthalmitis during hospitalization. Polymicrobial infections were present in twenty-one eyes, involving Staphylococcus aureus (*n* = 6), Staphylococcus epidermidis (*n* = 5), coagulase-negative Staphylococcus (*n* = 5), and Streptococcus pneumoniae (*n* = 3). Additionally, two patients had concurrent fungal keratitis.

The treatments performed for the patients (medical and surgical) are summarized in Table 2 (Table 2).

**Table 2:** Medical and surgical treatments of Moraxella keratitis

Topical antimicrobial medications	Number (percentage%)
*Empirical medications*	***106 (100)***
Amikacin + cefazolin	45 (42.5)
Amikacin + ceftazidime	37 (34.9)
Vancomycin + ceftazidime	11 (10.4)
Ofloxacin	11 (10.4)
Chloramphenicol	2 (1.8)
*Modified medications*	***31 (29.2)***
Levofloxacin	12 (11.3)
Ceftazidime	6 (5.7)
Ofloxacin	4 (3.8)
Amikacin + cefazolin	4 (3.8)
Amikacin + ceftazidime	3 (2.8)
Natamycin + voriconazole	1 (0.9)
Amphotericin B	1 (0.9)
Systemic medications*	79 (74.5)
Doxycycline	74 (69.8)
Ciprofloxacin	5 (4.7)
Surgical interventions	**50 (47.2)**
Cyanoacrylate glue and bandage contact lens	20 (18.9)
Tarsorrhaphy	19 (18.0)
Penetrating keratoplasty	9 (8.5)
Evisceration	2 (1.8)

*These antibiotics were combined with topical medications

In vitro antimicrobial sensitivity results are summarized in Table 3 (Table 3).

**Table 3:** In vitro antimicrobial sensitivity results

Antibiotic	Sensitive	Resistant	Sensitivity test not done
Ciprofloxacin	102	2	4
Amikacin	101	0	5
Ceftazidime	96	2	8
Gentamicin	97	1	8
Levofloxacin	89	8	9
Cefazolin	66	11	29
Imipenem	76	0	30
Chloramphenicol	61	2	43
Penicillin G	6	23	77
Co-trimoxazole	9	17	80
Tazocin	15	0	91
Ofloxacin	7	0	99

34% of patients had BCVA at hand motion, 21% at finger count, 23% light perception, and 9.4% no light perception. The mean baseline BCVA of the patients was 2.28 ± 0.6 LogMAR, while this amount reached 1.49 ± 0.81 LogMAR in the 6-month follow-up (P-value = 0.02).

17% of patients experienced corneal perforation at baseline and in the course of treatment. The prevalence of perforation did not differ significantly between diabetic and non-diabetic patients (*p* = 0.515).

Among the 86 patients without diabetes, 37 (43.0%) required surgical interventions: 17 received glue and bandage contact lens (BCL), 14 underwent tarsorrhaphy, 5 had penetrating keratoplasty, and 1 had evisceration. In comparison, 13 patients (65.0%) with diabetes needed surgical interventions: 3 received glue and BCL, 5 underwent tarsorrhaphy, 4 had penetrating keratoplasty, and 1 had evisceration. However, there was no significant difference between patients with and without diabetes (*p* = 0.076). Patients with hypertension (HTN) underwent surgical interventions more frequently than those without HTN (75% vs. 39%, *p* = 0.002). Among those without HTN, 61% recovered with only medication, while 32 needed surgical interventions: 16 received glue and BCL, 10 underwent tarsorrhaphy, and 6 had penetrating keratoplasty. Among those with HTN, 18 patients underwent surgeries: 4 received glue and BCL, 9 underwent tarsorrhaphy, 3 had penetrating keratoplasty, and 2 had evisceration.

All three factors - corneal perforation, HTN, and older age- are related to a higher need for surgical interventions. Corneal perforation is the most influential among these three predisposing factors, with an adjusted odds ratio of 19.2, followed by HTN with an adjusted odds ratio of 3.62 (Table 4).

**Table 4:** Risk factors of surgical interventions

Variables	Odds ratio	95% confidence interval		*P*-value
		Lower bound	Upper bound	
Age	1.038	1.010	1.068	0.008*
Hypertension	3.621	1.130	11.600	0.03*
Corneal perforation	19.276	3.543	104.881	0.001*

* Multinominal logistic regression

The mean BCVA in the six-month follow-up of the patients who needed surgical interventions was significantly lower than the patients who received only medical treatment (2.15 ± 0.65 vs. 1.29 ± 0.75 LogMAR, P-value = 0.02). However, the analysis regarding the antibiotics administered and the final BCVA after six months did not show any significant correlation (P-value = 0.44).

## Discussion

This study was designed to evaluate the clinical and bacteriological features of Moraxella keratitis in our tertiary eye center from 2015 to 2022. The most notable characteristics of Moraxella keratitis in this large series of patients were their severity of presentation, slow response to antimicrobials, high risk of surgical intervention, and poor BCVA, leading to blindness in nearly 9% of cases. HTN, corneal perforation, and advancing age were introduced as the risk factors for poor prognosis (need more surgical interventions) in this population. After controlling potential confounders, the multinomial logistic regression analysis presented that corneal perforation as the outstanding risk factor had the maximum strength of association with poor visual outcomes in Moraxella keratitis patients.

Moraxella catarrhalis has been identified as a potential cause of bacterial keratitis for more than a century [24]. It is known to cause severe infections, leading to a loss of vision [16]. Therefore, it is critical to be mindful of this bacterium’s potential role in such infections and to take appropriate precautions to ensure prompt and effective treatment. Due to its rarity, there is a scarcity of information about Moraxella keratitis in the current literature, despite significant research on its impact on other parts of the body. Previously, it was believed that up to 50% of cases of Moraxella keratitis were associated with risk factors such as diabetes and malnutrition brought on by persistent alcoholism [25]. It should be noted that these findings were based on a limited sample size of only 8 patients. A larger sample study reported lower rates of cases: 9.3% in diabetes patients and 4.7% in those with alcohol and drug use [19]. It is possible that the observed discrepancy can be attributed to the limited sample size.

Our data confirms, for the first time, that HTN is a predictive factor for Moraxella keratitis. In HTN, elevated blood pressure triggers a localized immune response through the release of cellular debris. In individuals with a genetic predisposition, this initial immune priming can cause a stronger immune response and lead to further organ damage [26]. Based on current evidence, it is believed that the epithelial sodium channel (ENaC) plays a crucial role in managing blood pressure by facilitating the transportation of water and sodium across membranes in the kidney tubules. This process causes the retention of sodium and water, leading to an imbalance in fluid levels. Activation of ENaC can also be influenced by extrarenal factors such as reactive oxygen species (ROS) [27]. ROS plays an essential role in immune signaling pathways and is incorporated into immune responses, under regulated conditions [28, 29]. The overproduction of ROS poses a significant threat to vascular health. This phenomenon is primarily attributed to the heightened activity of reduced nicotinamide adenine dinucleotide phosphate (NADPH) oxidase, which is the principal enzyme responsible for converting oxygen to superoxide [30, 31]. Importantly, some evidence hypothesized that some immunodeficiency conditions such as acquired immune deficiency syndrome (AIDS) affect renal ENaC salt handling and salt-sensitive hypertension due to evidence of generating ROS [27]. It appears that this mechanism contributes to immunocompromised conditions in individuals with HTN, thereby increasing the risk of Moraxella keratitis.

Worldwide, Moraxella keratitis is a common cause of bacterial corneal ulcers that corresponds to 2 to 3% of all corneal ulcers [32]. Accurate data on the prevalence of this disease in different sexes are not yet available, but the current study showed that more than 80% of patients with Moraxella keratitis are men. Even though sex has not been investigated in humans for the possibility of Moraxella keratitis infection, men are more likely to contract the disease compared to women, possibly due to occupational exposures.

The Irish series reports that 68% of the patients had local risk factors that compromised the immunity of the ocular surface. These risk factors included blepharitis and dry eye (38%), prior surgery (30%), and topical corticosteroid usage (16%) [33]. It was found that almost 50% of the patients had systemic risk factors, with diabetes mellitus (19%), chronic alcohol abuse (16%), and thyroid disease (11%) being the most prevalent ones [33]. The aforementioned findings align with other studies that have identified diabetes mellitus and chronic alcohol misuse as significant risk factors [19, 34–36]. In this series, we demonstrated that HTN and advancing age were the best indicators of a poor outcome after corneal perforation in patients with Moraxella keratitis.

The study included patients with a mean age of 54 years, and more than half of them were over 50 years old. The results presented in the study conducted by Das et al. align with our findings indicating that a significant proportion of culture-proven Moraxella keratitis cases were observed in the elderly and middle-aged population [24]. This suggests that Moraxella species tend to affect immunocompromised individuals, highlighting their opportunistic nature.

The antimicrobial sensitivity results from our study indicate high susceptibility of Moraxella species to commonly used antibiotics such as ciprofloxacin (98%), amikacin (100%), ceftazidime (98%), gentamicin (99%), and levofloxacin (89%). This aligns with findings from previous studies, which also reported high sensitivity to fluoroquinolones and aminoglycosides [19, 37]. However, our study noted lower sensitivity to cefazolin (66%) and chloramphenicol (61%), contrasting with a study by Das et al. that reported higher efficacy of these antibiotics [24]. Notably, penicillin G and co-trimoxazole showed low sensitivity in both our study and the literature, reflecting the intrinsic resistance patterns of Moraxella species [38]. Imipenem displayed 100% sensitivity in our study, in line with Hoarau et al.‘s study, which also observed excellent efficacy of imipenem against Moraxella [32]. These comparisons underscore the importance of local antimicrobial susceptibility testing to guide the effective treatment of Moraxella keratitis, as regional variations in resistance patterns may influence therapeutic outcomes.

This study has several key limitations. The assessment of outcomes was not conducted using an objective grading system, which resulted in information bias. We would like to bring to your attention that our study was conducted retrospectively, which may have led to certain biases and limitations in data collection. On the other hand, due to the location of our hospital in the capital of Iran and its referral status, patients continued their treatment in nearby cities, so only 6-month follow-ups of patients were available and other follow-ups were missed. The study’s small sample size poses a significant limitation.

Future research should include prospective studies with larger sample sizes and objective grading systems to validate findings, along with long-term follow-up to understand treatment efficacy. Investigating hypertension’s role and mechanisms like ENaC and ROS in Moraxella keratitis is essential. Developing preventive strategies for high-risk groups and conducting global, sex-based studies to compare prevalence and outcomes are also important. Additionally, exploring novel therapeutics and combination therapies to combat antimicrobial resistance and improve patient outcomes is crucial.

## Conclusion

Based on our experience, it is evident that ocular predisposing factors play a vital role in causing Moraxella keratitis which can lead to severe corneal infection and may require surgical interventions. HTN, corneal perforation, and advancing age were identified as risk factors for poor prognosis requiring surgical interventions in this population. Therefore, it is crucial to take necessary precautions and preventive measures to avoid such infections in the future.

## Data Availability

On request from the corresponding author, data supporting the findings of this study will be made available.

## Abbreviations

MK: Microbial keratitis
BCVA: Best-corrected visual acuity
HTN: Hypertension
WHO: World Health Organization
PKP: Penetrating keratoplasty
LP: Light perception
NLP: No light perception
SD: Standard deviation
IQR: Interquartile range
ENaC: Epithelial sodium channel
ROS: Reactive oxygen species
NADPH: Nicotinamide adenine dinucleotide phosphate
AIDS: Acquired immune deficiency syndrome
